# Involvement of the Gap Junction Protein, Connexin43, in the Formation and Function of Invadopodia in the Human U251 Glioblastoma Cell Line

**DOI:** 10.3390/cells9010117

**Published:** 2020-01-03

**Authors:** Amandine Chepied, Zeinaba Daoud-Omar, Annie-Claire Meunier-Balandre, Dale W. Laird, Marc Mesnil, Norah Defamie

**Affiliations:** 1 Equipe 4CS, Laboratoire Signalisation et Transports Ioniques Membranaires (STIM), CNRS ERL 7003, Pôle Biologie Santé, University of Poitiers, 86073 Poitiers, France; amandine.chepied@univ-poitiers.fr (A.C.); zeinaba.daoud.omar@etu.univ-poitiers.fr (Z.D.-O.); annie-claire.balandre@univ-poitiers.fr (A.-C.M.-B.); marc.mesnil@univ-poitiers.fr (M.M.); 2 Department of Anatomy and Cell Biology, Schulich School of Medicine and Dentistry, The University of Western Ontario, London, ON N6A 3K7, Canada; Dale.Laird@schulich.uwo.ca

**Keywords:** cell invasion, connexin, gap junction, glioblastoma, invadopodia

## Abstract

The resistance of glioblastomas to treatments is mainly the consequence of their invasive capacities. Therefore, in order to better treat these tumors, it is important to understand the molecular mechanisms which are responsible for this behavior. Previous work suggested that gap junction proteins, the connexins, facilitate the aggressive nature of glioma cells. Here, we show that one of them—connexin43 (Cx43)—is implicated in the formation and function of invadopodia responsible for invasion capacity of U251 human glioblastoma cells. Immunofluorescent approaches—combined with confocal analyses—revealed that Cx43 was detected in all the formation stages of invadopodia exhibiting proteolytic activity. Clearly, Cx43 appeared to be localized in invadopodia at low cell density and less associated with the establishment of gap junctions. Accordingly, lower extracellular matrix degradation correlated with less mature invadopodia and MMP2 activity when Cx43 expression was decreased by shRNA strategies. Moreover, the kinetics of invadopodia formation could be dependent on Cx43 dynamic interactions with partners including Src and cortactin. Interestingly, it also appeared that invadopodia formation and MMP2 activity are dependent on Cx43 hemichannel activity. In conclusion, these results reveal that Cx43 might be involved in the formation and function of the invadopodia of U251 glioblastoma cells.

## 1. Introduction

The most common brain tumor in adults, glioblastoma, is characterized by a low 5-year survival (less than 3% for primary glioblastoma or ~10% for secondary glioblastoma) [1]. Such a low curability is mostly the consequence of glioblastoma cells disseminating and infiltrating widely within the brain, rendering the tumor resection incomplete. Therapy is further hindered by the resistance of these infiltrating cells to chemo- and radio-therapies. Therefore, elucidating the molecular mechanisms responsible for glioblastoma invasion is necessary to develop more adapted therapeutic targets.

During the last decade, several in vitro studies revealed that the expression of the gap junction protein, connexin43 (Cx43), could favor migration and invasion of brain tumor cells [2,3,4]. Similar observations were not only obtained in the glioma context but also during development since Cx43 seems necessary for cortical migration of neuron precursors [5] and neural crest cells [6,7,8]. Paradoxically, Cx43 was initially considered a tumor suppressor, as glioma cell expression of Cx43 decreased their proliferation in vitro and in vivo [9,10,11]. Such observations conferred to Cx43-opposing roles in glioma cells; acting as a tumor suppressor by decreasing cell growth in one context and acting as an oncogene by increasing invasion capacity in another [12]. How Cx43 mediates such contradictory effects is unknown, although data obtained with rat C6 glioma cells and neuron precursors provided a hint that suggests that regulation of migration and invasion is linked to protein interactions that occur at the Cx43 carboxy terminus (Ct) [13,14]. These results led to the hypothesis that the intracellular Ct domain of Cx43 mediates the rearrangement of actin cytoskeleton, thus affecting cell migration [15,16]—even if Cx43 hemichannels were also suggested to have an effect on migration as shown for neurite outgrowth [17].

The actin cytoskeleton is involved in the invasion process by playing a key role in focal adhesions (FAs) [18,19] and invadopodia formation and function [20,21]. FAs permit adhesion to extracellular matrix (ECM) components and invadopodia permit their degradation. FAs are characterized by integrin clusters connected to actin fibers, while invadopodia are membrane protrusions which form as a consequence of rapid actin polymerization [22,23]. Tumor cell invasion is a consequence of coordinated mechanisms acting on FA turnover and digestive processes favored by invadopodia formation. Such coordination is possible through a common set of proteins that temporally and spatially act on FAs formation and invadopodia growth [24]. One of these proteins is Src, which interacts with the focal adhesion kinase (FAK) for activating integrins and adhesion [25,26,27]. Apparently, such an interaction prevents Src from engaging in invadopodia formation [28]. When Src is released from FAs, it phosphorylates key components (cortactin, N-WASP, cofilin) required for invadopodia formation, which is initiated by recruiting the scaffold protein TKS5 [28,29]. Several of the key actors modulating invadopodia growth (cortactin, Src, cofilin) are known to interact with the Ct domain of Cx43 [30,31]. However, despite these observations, the molecular mechanisms linking Cx43 to tumor cell invasion remain obscure. 

To better understand the role played by Cx43 in invasion, we used as a model of human glioblastoma cells, the U251 cell line. This cell line was chosen because it endogenously expresses Cx43 and is invasive [32]. To circumvent limitations associated with the overexpression Cx43 to assess the role of Cx43 in tumorigenesis, we used shRNAs to downregulate its expression. In this study, we show for the first time that Cx43 is localized in invadopodia and is a component of the protein complex involved in their formation and function. Upon Cx43 knockdown in U251 cells, the number and length of invadopodia were decreased. Our data suggest that Cx43 acts on invadopodia through its interactome by bringing together the molecular components (src, cortactin) necessary for their formation but not through its hemichannel activity which negatively regulates the kinetics of their formation and function.

## 2. Materials and Methods

### 2.1. Antibodies

Monoclonal antibodies directed against Cx43 (BD Transduction Laboratories, NJ, USA), cortactin, MT1-MMP (Millipore, Billerica, USA) and GAPDH (HyTest, Turku, Finland) were used as well as polyclonal antibodies directed against FAK, P-FAK pY^397^, P-cortactin pY^466^, Src (Santa Cruz Biotechnology, Dallas, TX, USA), P-Src pY^416^ (Cell Signaling, Danvers, MA, USA) and TKS5 (Millipore). Anti-mouse and anti-rabbit secondary antibodies were conjugated to HRP (DakoCytomation, Glostrup, Denmark) for Western blot or to Alexa Fluor^®^ antibodies (Molecular Probes, Eugene, OR, USA) for immunofluorescence. Fluorescent labelling of actin cytoskeleton was performed by using TRITC-conjugated phalloidin (Sigma–Aldrich, Saint-Louis, MO, USA).

### 2.2. Cell Culture Conditions

Human U251 glioblastoma cell line and its two shRNA derived Cx43 knockdown clones (shRNA 1 and shRNA 2 clones) [32] were cultured in high glucose (4.5 g/L) Dulbecco’s Minimum Essential Medium (DMEM, Lonza, Brussels, Belgium) supplemented with fetal bovine serum (FBS; 10%, Lonza), l-glutamine (0.5 mM, Sigma–Aldrich) and antibiotics (100 U/mL penicillin, 100 µg/mL streptomycin, Lonza). Cultures were maintained in a water-saturated incubator (37 °C, 5% CO_2_). Routine cultures were kept under selection pressure (1 µg/mL of puromycin) to maintain an adequate Cx43 knockdown. Moreover, in order to limit the number of passages, clones were initially expanded during their selection to have sufficient stock to perform the scope of studies. Results were obtained from cells that had not reached passage 10. When specified, Petri dishes were coated with gelatin (0.2%) in PBS for one hour at room temperature before seeding cells. In some cases, cells were treated for 1 h with PP2 (10 mM) in DMSO (Calbiochem, Darmstadt, Germany)—a Src inhibitor, or flufenamic acid (FFA; 100 µM; Sigma–Aldrich)—a connexin channel inhibitor [33].

### 2.3. Quantitative Real-Time PCR

Total RNA was isolated using an SV Total RNA isolation kit according to the instructions of the manufacturer (Promega, Charbonnière-les-Bains, France). Briefly, the total RNA (1 mg) was reversely transcribed using Superscript II (Invitrogen, Paris, France). The reactions of qPCR were performed in 20 mL total volume with 2.5 pmol of each primer, 10 mL of SYBRgreen Master Mix 2X (Applied Biosystems, Warrington, UK) and 0.5 mL of template. The amplification and analysis were performed using an ABI Prism 9500 Sequence Detection System (Applied Biosystems). Samples were compared using the relative CT method. Specific primers for Cx43 and housekeeping gene (GAPDH) were synthesized by Eurogentec (Liège, Belgium): (1) Cx43 (BC026329): mCx43-forward, 5′-GTG-CCG-GCT-TCA-CTT-TCA-TTA-AG-3′; mCx43-reverse, 5′-CCA-AGG-CGC-TCC-AGT-CA-3′ and (2) GAPDH (NM002046): GAPDH-forward, 5′-TGC-ACC-ACC-AAC-TGC-TTA-GC-3′; GAPDH-reverse, 5′-GGC-ATG-GAC-TGT-GGT-CAT-GAG-3′.

### 2.4. Western Blot

Proteins were subjected to electrophoretic separation using 10% polyacrylamide-SDS gel (InVitrogen) and transferred to nitrocellulose membrane (GE Healthcare, Buckinghamshire, UK). Membranes were blocked in 1 M Tris-buffer saline solution (pH 8.0) containing Tween 20 (0.1%) and non-fat dry milk (5%) for 3 h at room temperature. Blots were then incubated overnight at 4 °C with monoclonal antibodies against Cx43 (1:1000), cortactin (1:1000), GAPDH (1:40,000) and MT1-MMP (1:1000) or polyclonal antibodies against P-cortactin (1:1000), total Src (1:1000), Y416 P-Src (1:1000), FAK (1:1000), P-FAK (1:1000) and TKS5 (1:1000), followed by detection using HRP-conjugated antibodies (DakoCytomation) and an enhanced chemiluminescence kit (ThermoScientific, IL, USA). Densitometric analyses were performed using ImageJ software.

### 2.5. Fluorescent-Gelatin Degradation Assay

Sterilized coverslips were coated with 0.01% (*w*/*v*) poly-l-lysine solution (Sigma) for 40 min at room temperature, washed with PBS, and fixed with 0.5% glutaraldehyde (Sigma, Saint-Louis, USA) for 15 min, followed by extensive washing. Then, coverslips were inverted on a 30 µL drop of fluorescent Oregon Green^®^ 488-conjugated gelatin (FG-gelatin; Molecular Probes, Eugene, USA) and incubated at room temperature for 10 min. For inserts, 30 µL of FG-gelatin (0.2%) was added on for 10 min, 20 µL gelatin was removed and the inserts were put at 37 °C for 1 h. Once rinsed with PBS, the residual reactive groups in gelatin were quenched with sodium borohydride (5 mg/mL) for 5 min, followed by extensive washing with PBS.

To assess the ability of cells to form invadopodia and to degrade matrix, the cells were plated on coverslips (15 × 10^3^ cells/mL) or inserts (10^4^ cells/mL) coated with fluorescent FG-gelatin in 24-well plates and maintained in a water-saturated incubator (37 °C, 5% CO2). After this preparation, indirect immunofluorescence was performed [29].

### 2.6. Invadopodia Synchronization

U251 cells were cultured on FG-gelatin (Molecular Probes) for 24 h in the presence of 5 µM BB94 (Santa Cruz) to block metalloproteases and invadopodia formation. BB94 was then washed out to allow synchronous invadopodia formation [34].

### 2.7. Indirect Immunofluorescence

The immunodetection of Cx43, cortactin, P-FAK, TKS5 and MT1-MMP was performed on cells (U251 mock and shRNA clones) present on coverslips or inserts coated with FG-gelatin as described previously. F-actin was detected using TRITC-phalloidin. The cells were fixed in paraformaldehyde (4%) for 20 min at room temperature. After incubation in a blocking solution (2% bovine serum albumin, 1% Triton X-100 in PBS), the cells were incubated with primary antibodies (1:250) overnight at 4 °C. Alexa Fluor^®^ 555- or 647-conjugated antibodies (1:250, Invitrogen) and/or TRITC-phalloidin (1:50) were then applied on the preparations for 1 h. Coverslips or inserts were mounted afterwards with Mowiol (Calbiochem, Darmstadt, Germany) prior to observation with confocal microscopy.

### 2.8. Confocal Fluorescence Microscopy

In our confocal microscopy conditions, only three fluorescent labelings were available as green (488 nm), visible red (545 nm) and deep invisible red (633 nm). Because of this limitation, the detection of Cx43 in invadopodia was assessed as follows. As a first step, the presence of invadopodia was checked at the level of digested areas of green-fluorescent gelatin (FG-gelatin; 488 nm) which appear as black spots. The second step was to identify at the level of the black spots of invadopodia through their molecular components (actin or cortactin), which were detected by visible red labeling (545 nm). Thereafter, the third step was the detection of Cx43 by deep invisible red labeling (633 nm) which was converted in white for visualization.

Confocal images were obtained using an Olympus IX81 laser scanning confocal inverted microscope with 40X UAPO ID/340UV NA 135 oil or 60X O.N. 1.4 PLAPO oil objectives. Images were processed with FluoView software.

### 2.9. Co-Immunoprecipitation

In presence of antibodies against Cx43, 200 µg of protein from each cell type (U251 mock and shRNA clones) was incubated in RIPA buffer (150 mM NaCl; 50 mM Tris-HCl, pH 7.4; 1 mM EDTA; 1% NP40; 1 mM Na_3_VO_4_; 50 mM NaF) containing a protease inhibitor cocktail (Sigma–Aldrich) overnight at 4 °C. The immune complexes were collected by incubating the mixture with 50 µL of protein G-sepharose beads for 2 h. Non-specifically bound proteins were removed by washing the beads 5 times in 1mL RIPA buffer after centrifugation. Bound material was then solubilized in 30 µL of two-fold concentrated Laemmli sample buffer and boiled for 5 min. Then, samples were analyzed by Western blot.

### 2.10. Zymography

In Petri dishes containing confluent cells, the culture medium was replaced for 4 h with FBS-free medium. Then, FBS-free medium was changed for 24 h. Culture medium was collected, centrifuged and concentrated. Proteins were quantified and loaded in a migration gel (1.5 mm thickness; 10% polyacrylamide-SDS; 0.3% gelatin) for 1.5 h at 120 V. The gel was incubated 3 times in water containing 2.5% Triton-X-100 for 1 h and washed with incubation buffer (50 mM Tris; 10 mM CaCl_2_; 150 mM NaCl; 2.5 µM ZnCl_2_; 0.05% Brij 35; pH 7.5) for 20 min and left in this buffer for 48 h at 37 °C. Gel was stained for 1 h with Coomassie blue (10% acetic acid; 30% methanol; 0.5% Coomassie blue) and discolored (4% acetic acid, 8% methanol) 5 times for 20 min each. Lysis bands were observed and quantified using ImageJ software.

### 2.11. Dye Uptake Assay

One hour before experimentation, if necessary, 100 µM FFA were added in culture medium of Petri dishes or in the bottom compartment of the Boyden chamber in inserts. After 10 min, the FFA was washed out and U251 cells were incubated at 37 °C for 10 min in a calcium-free solution (extracellular solution containing 10 mM EGTA; pH 7.4). The dye tracer Lucifer Yellow (457 kDa) was added (0.5 mg/mL) to the solution for 5 min. Cultures were then washed four times with a calcium-free solution. Samples were observed with a fluorescence microscope (MacroView MVX10 Olympus, Rungis, France) [35].

### 2.12. Statistical Analyses

All experiments were repeated independently, at least three times. Statistical analyses were performed using GraphPad Prism 4 software. All reported data are expressed as mean ± SEM and significant differences were identified by unpaired Student’s *t*-test or One-way Anova. * *p* < 0.05; ** *p* < 0.01; *** *p* < 0.001.

## 3. Results

### 3.1. U251 Cells form Invadopodia

**Figure 1:**
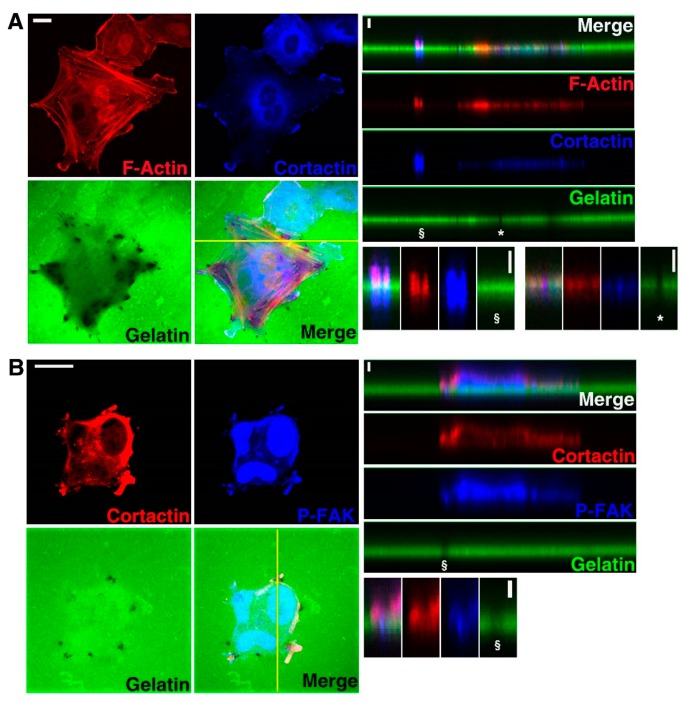
U251 cells form invadopodia. Cells were cultured on coverslips coated with FG-gelatin (1.5 × 10^4^ cells/mL) and observed by confocal microscopy in the *xy* dimension. After 5 h, the localization of (**A**) F-actin (Red) and cortactin (Blue) or (**B**) cortactin (Red) and P-FAK (Blue) was determined by indirect immunofluorescence or using TRITC-phalloidin. Invadopodia formation (*) and focal adhesions (§) were observed. Each left panel is *xy* images, right top panel is *xz* images and right bottom panel is a *xz* enlargement of the regions of interest (N = 10). The yellow line in the *xy* images is the axis shown in the *xz* dimension (Scale bar: 20 µm on *xy* dimension and 2 µm on *xz* dimension).

In order to assess if U251 cells develop invadopodia and degrade ECM, they were seeded on FG-gelatin. Five hours later, black areas of digested gelatin became visible underneath cells as observed by confocal microscopy (Figure 1). At most of these gelatin-depleted areas, two components enriched in invadopodia—cortactin and F-actin—were detected, revealing these invasive structures as ventral protrusions of U251 cells (Figure 1A). Moreover, the colocalization of cortactin with the membrane-associated type-I transmembrane MMP (MT1-MMP) or TKS5 in areas of gelatin degradation, confirmed these structures as invadopodia (Figure S1A,B). Since studies showed that invadopodia and FA share common components (actin, cortactin), we specifically looked for the presence of FAK as a surrogate for positioning FA. The presence of FA in U251 cells was indeed demonstrated by detecting the activated, phosphorylated form of FAK (P-FAK) in zones distinct from matrix degradation where cortactin was not expressed (Figure 1B). Moreover, the fact that P-FAK was colocalized with cortactin and associated with gelatin degradation at the leading edge of cells suggested they were constituents of lamellipodia necessary for cell migration (Figure 1B).

### 3.2. Cx43 Is A Component of Invadopodia

**Figure 2:**
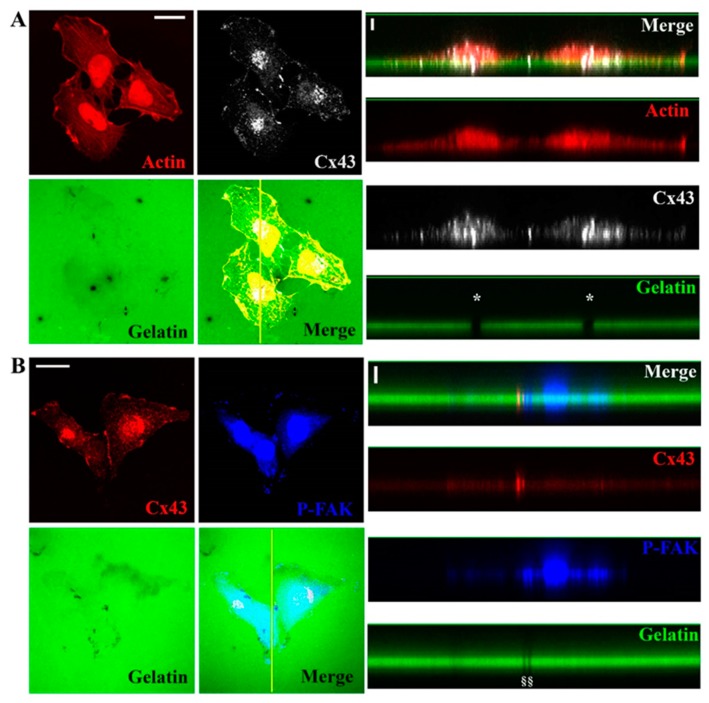
U251 cells form invadopodia containing Cx43. Cells were cultured on coverslips coated with FG-gelatin (1.5 × 10^4^ cells/mL) and observed by confocal microscopy in the *xy* dimension. After 5 h, localization of (**A**) F-actin (Red) and Cx43 (White) or (**B**) Cx43 (Red) and P-FAK (Blue) was determined by indirect immunofluorescence or using TRITC-phalloidin. Invadopodia formation (*) and focal adhesions (§) were observed. Each left panel is *xy* images, the right top panel is *xz* images and the right bottom panel is an *xz* enlargement of the regions of interest (N = 10). The yellow line in the *xy* images is the axis shown in the *xz* images (Scale bar: 20 µm on *xy* dimension and 2 µm on *xz* dimension).

Since Cx43 was suggested to support cancer cell invasiveness [5,6,7,8], its presence and localization was assessed in U251 cells seeded on FG-gelatin. Cx43 appeared to be localized in ventral protrusions at the location of digested areas where F-actin accumulated (Figure 2A). Moreover, we observed that Cx43 was also colocalized with cortactin and TKS5 (Figure S2A,B). In contrast, Cx43 was not colocalized with P-FAK and sites of cell–cell apposition (Figure 2B). As such, it appears that Cx43 could represent a marker of invadopodia and not of FAs.

**Figure 3:**
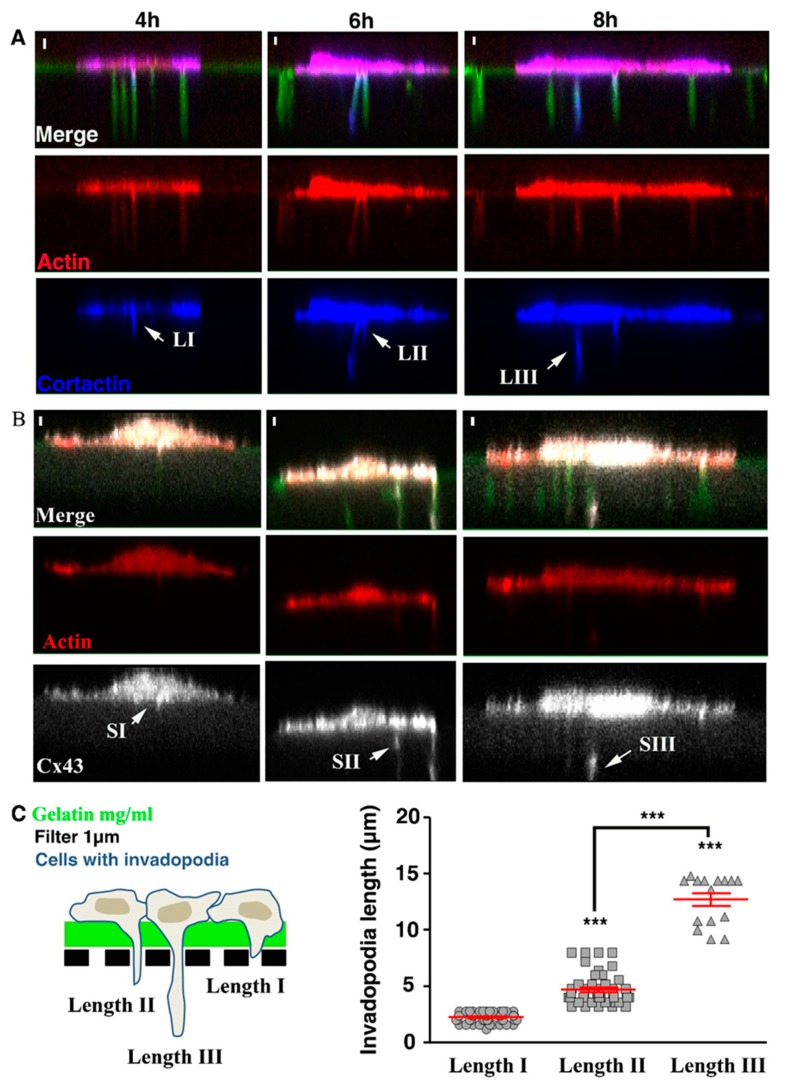
Cx43 is localized in invadopodia during their fomation and maturation. (**A**,**B**) Cells were cultured (1 × 10^4^ cells/mL) on inserts (1 µm porosity), coated with FG-gelatin allowing invadopodia formation and maturation. Filters from inserts were observed by confocal microscopy in xz dimension at 4 h (left panel), 6 h (middle panel) or 8 h (right panel) after seeding cells. (**A**) F-actin (Red) and cortactin (Blue) or (**B**) F-actin (Red) and Cx43 (White) were observed by indirect immunofluorescence or using TRITC-phalloidin. Three stages of invadopodia formation and maturation were determined according to invadopodia length and time of culture: Initiation Stage (SI; Length I: LI) at 4 h, Assembly Stage (SII; Length II: LII) at 6 h, and Elongation/Maturation Stage (SIII; Length III: LIII) at 8 h (N = 10) (Scale bar: 2 µm). (**C**) Schematic representation of insert experiment with the three stages of invadopodia formation and maturation on left panel. Right panel represents the observed invadopodia length (µm) according to the stages of invadopodia formation and maturation (LI (N = 40): 2.24 ± 0.06 µm; LII (N = 40): 4.69 ± 0.2 µm and LIII (N = 15): 12.69 ± 0.56 µm). *** *p* < 0.001. Values shown as mean ± SEM.

Formation of invadopodia can be divided into three stages: initiation/invadopodium precursor (stage I), assembly/polymerization stage (stage II) and maturation/mature invadopodium (stage III) [24,36]. To distinguish these stages, U251 cells were seeded on filters (pores of 1 µm diameter) coated with FG-gelatin (Figure 3C, scheme). Through the colocalization of F-actin and cortactin, invadopodia development was revealed across the 1µm-pores of the filter (Figure 3A). We distinguished 3 different lengths of active invadopodia, called lengths I, II or III which correlate to the stages described above (Figure 3C, right panel). Invadopodia appeared 4 h after seeding the cells (length I) while lengths II and III were observed 6 and 8 h later, respectively (Figure 3A). Using confocal microscopy, Cx43 was detected at the base of length I structures and mostly at the tip of the invadopodia at lengths II and III (Figure 3B).

### 3.3. Cx43 Interacts with Key Proteins in Formation of Invadopodia

**Figure 4:**
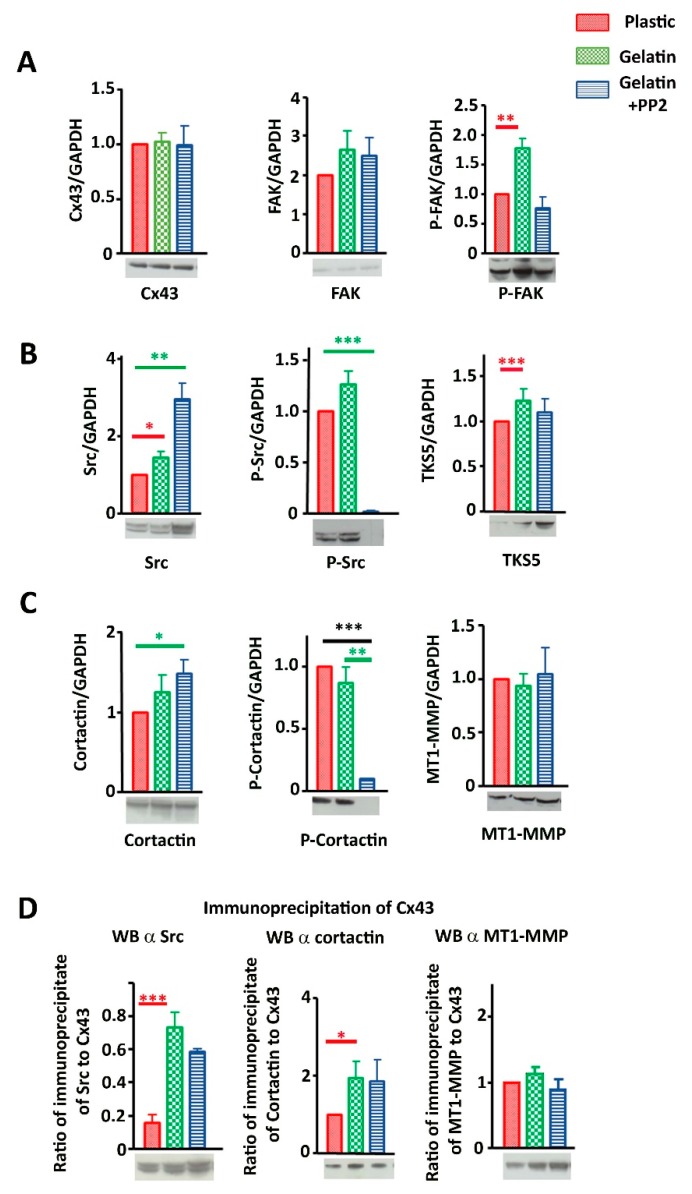
Protein (Cx43, FAK, P-FAK, Src, P-Src, cortactin, P-cortactin, TKS5 and MT1-MMP) expressions and interactions in correlation with Cx43 expression. (**A**) Densitometric analysis of Cx43 (left), FAK (middle) and P-FAK (right) expression levels after normalization by GAPDH expression. (**B**) Densitometric analysis of Src (left), P-Src (middle) and TKS5 (right) protein expression level after normalization to GAPDH levels according to Cx43 expression level (N = 5). (**C**) Densitometric analysis of cortactin (left), P-cortactin (middle) and MT-MMP (right) protein expression levels after normalization by GAPDH expression (N = 4). (**D**) Ratio of immunoprecipitation of Cx43 from U251 cells and immunoblotting of Src (N = 3), immunoblotting of cortactin (N = 5) or immunoblotting of MT1-MMP (N = 5). Red bar highlights differences due to gelatin culture condition and black bar highlights differences due to Cx43 expression diminution. Green bar highlights differences due to PP2 exposition. * *p* < 0.05; ** *p* < 0.01 and *** *p* < 0.001. Values shown as mean ± SEM (see Supplementary Table S1).

Knowing that Cx43 interacts with many partners through its Ct domain, we therefore tested for the expression of some important proteins in the formation process of the invadopodia, and their possible interaction with Cx43. Since Src-dependent phosphorylation modulates Cx43 function (found in invadopodia but not in FA) and FAK (found in FA but not in invadopodia), we were interested to compare their respective amount (Cx43, FAK and Src) in U251 cells when cultured in conditions permitting invadopodia formation (gelatin) or not (plastic). By Western blot, it appeared that those conditions did not affect Cx43 levels, even in the presence or absence of PP2, a Src activity inhibitor (Figure 4A, Cx43). Similar results were found for FAK (Figure 4A), P-Src (Figure 4B), cortactin (Figure 4C) and MT1-MMP (Figure 4C), except for P-FAK, Src and TKS5, whose levels were higher when cells were cultured on gelatin compared to plastic (Figure 4A, P-FAK; Figure 4B, Src and TKS5). Thus, in conditions permitting invadopodia formation (gelatin_coated dish), an increase in specific protein expression was observed (Figure 4A, P-FAK; Figure 4B, Src and TKS5).

In parallel, by performing co-immunoprecipitation, we showed that Src/Cx43 and cortactin/Cx43 interactions were increased when cells were seeded on gelatin (Figure 4D). However, no difference was observed for MT1-MMP/Cx43 interaction in the different culture conditions (Figure 4D, right). Furthermore, co-immunoprecipitation revealed no interaction between Cx43 and P-Src, FAK, P-FAK, P-cortactin or TKS5 (data not shown). Taken together, these results suggest that Cx43 acts on invadopodia growth by interacting with partners such as Src and cortactin. Indeed, on plastic, when invadopodia formation is not permitted, Cx43/Src or cortactin interactions were weak (Figure 4D; left and middle). On the contrary, when invadopodia formation is stimulated by gelatin, these interactions become significantly increased (Figure 4D; left and middle). Since Src and cortactin are known to be key proteins involved in invadopodia formation, Cx43 interaction with these two key proteins is probably essential for invadopodia formation.

Interestingly, Src and cortactin expression were increased and P-Src or P-cortactin was decreased on gelatin when Src autophosphorylation was inhibited by PP2 (Figure 4B, Src and P-Src; C, P-cortactin). Contrastingly, no effect of PP2 was found on P-FAK (Figure 4A, P-FAK).

As it has been already shown in the process of invadopodia formation [28,29], Src autophosphorylation—needed for its activation and cortactin phosphorylation—is not involved in Cx43 expression nor in its interaction with Src and cortactin.

Therefore, Cx43 could act also as an interacting protein, as shown by its localization in invadopodia (Figure 3B). This assumption was even confirmed by cultivating cells at different densities. When cells were grown at high densities that promoted gap junction plaque formation, less invadopodia were observed (Figure S3A,C). These results suggest that gap junctions and invadopodia formation would depend on culture densities (Figure S3B).

### 3.4. Cx43 Knockdown Is Associated with Reduced Gelatin Degradation

**Figure 5:**
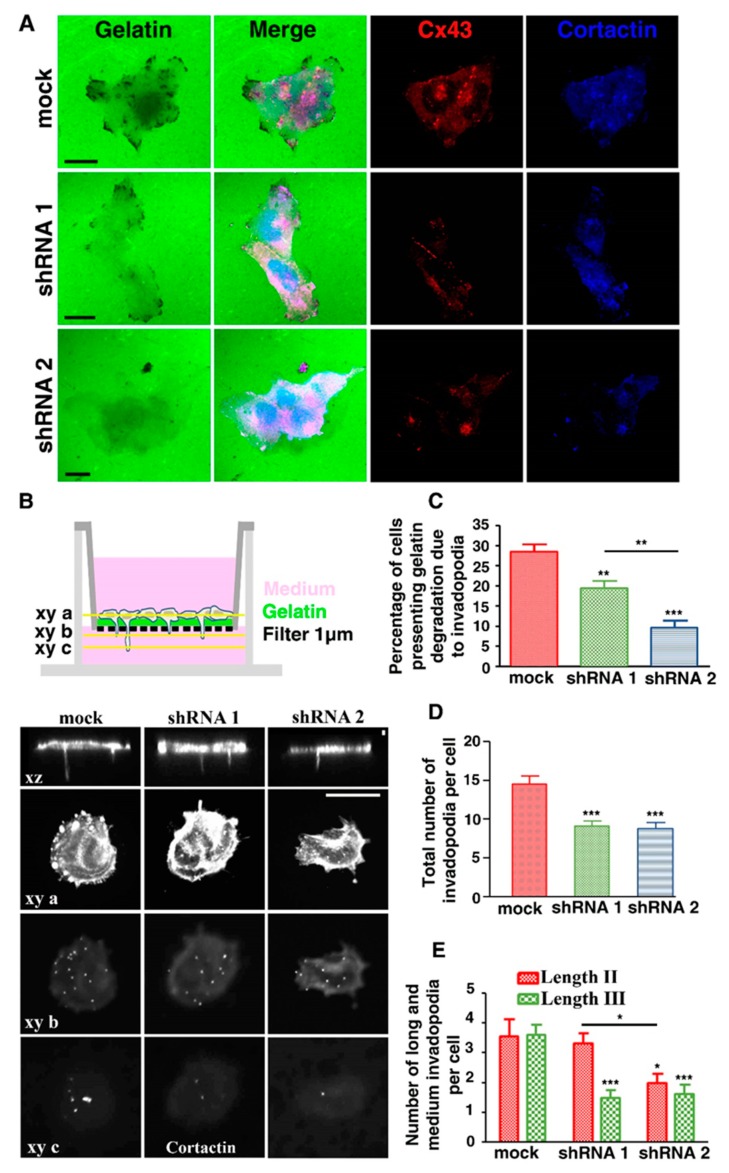
Invadopodia formation and maturation depend on Cx43 expression level. (**A**) U251 mock cells (top) or shRNA 1 (middle) and 2 (bottom) cells were cultured on coverslips coated with FG-gelatin (1.5 × 10^4^ cells/mL) and observed by confocal microscopy in *xy* dimension. (**A**) After 5 h, localization of Cx43 (Red) and cortactin (Blue) was determined by indirect immunofluorescence on coverslips in the three U251 clones (N = 10), (Scale bar: 20 µm). (**B**) Schematic representation of the experiment that was performed in bottom panel. Three different levels of observation were determined (*xy a* slice corresponds to cell body; *xy b* slice was just below insert and corresponds to Assembly stage of invadopodia formation; *xy c* slice was 4.5 µm underneath the insert and corresponds to Elongation/Maturation stage of invadopodia formation). The bottom panel presents a confocal microscopy of the three U251 clones cultured on inserts coated with FG-gelatin. Cortactin (White) was detected by indirect immunofluorescence. Cells were observed in the *xz* dimension (top) or *xy a, b* or *c* dimensions (bottom), (N = 10), (Scale bar: 20 µm on *xy* dimension and 2 µm on *xz* dimension). (**C**) Percentage of cells presenting gelatin degradation due to the presence of invadopodia was determined according to Cx43 expression level in U251 mock cells and shRNA clones. (**D**) Total number of invadopodia per cell was determined according to Cx43 expression level. (**E**) Number of long and medium invadopodia per cell was determined according to Cx43 expression level. Medium invadopodia correspond to Assembly stage of invadopodia formation (Stage II/Length II, *xy b* slice) and long invadopodia correspond to Elongation/Maturation stage of invadopodia formation (Stage III/Length III, *xy c* slice). * *p* < 0.05; ** *p* < 0.01 and *** *p* < 0.001. Values shown as mean ± SEM (see Supplementary Table S1).

The presence of Cx43 at different lengths of invadopodia formation suggests that it may be involved in their formation and growth. To verify if Cx43 is really involved in the formation of invadopodia, two Cx43 shRNA clones of U251 cells (shRNA 1 and shRNA 2) exhibiting decreased Cx43 expressions (60% and 75%, respectively) were used (Figure S5) [32]. When seeded on FG-gelatin, both clones showed a reduction in their ability to digest FG-gelatin (Figure 5A). The capacity of Cx43 shRNA cells to degrade FG-gelatin was 1.5–3 times less than the mock cells (Figure 5C). Moreover, immunofluorescence confirmed that Cx43 expression was decreased in both Cx43 knockdown clones (Figure 5A), while cortactin localization was not changed. These results show that decreased FG-gelatin degradation is correlated with inhibition of Cx43 expression. With MT1-MMP being detected in invadopodia of U251 cells (Figure S1A), we tested matrix metalloproteinase 2 (MMP2) activity, which depends on MT1-MMP, by zymography. This approach revealed that MMP2 activity was slightly decreased in Cx43 knockdown cells compared to controls (Figure S4).

### 3.5. Cx43 Knockdown Is Associated with Decreased Invadopodia Formation and Growth

In addition to Cx43 knockdown resulting in decreased FG-gelatin digestion, there was a significant reduction in the number of invadopodia per shRNA cell compared to mock cells (Figure 5D). To assess if the stage of invadopodia formation was affected by reduced Cx43 expression, 8 h after seeding cells on gelatin-coated filters, the number of invadopodia reaching lengths II (slice xy b) and III (slice xy c) were quantified (Figure 5B, bottom panel). The mock cells exhibited the highest number (3 to 4) of invadopodia per cell for both lengths (Figure 5E). Cx43 knockdown cells (shRNA 1) exhibited a similar number of length II invadopodia but half (two per cell) length III compared to mock cells while shRNA 2 cells exhibited a low number (two per cell) of lengths II and III invadopodia (Figure 5E). Therefore, the reduced number of invadopodia was related to decreased Cx43 expression in U251 cells and supports the hypothesis that Cx43 is involved in their formation and growth.

### 3.6. Cx43 Facilitates Formation and Growth of Invadopodia by Interacting with Key Proteins

**Figure 6:**
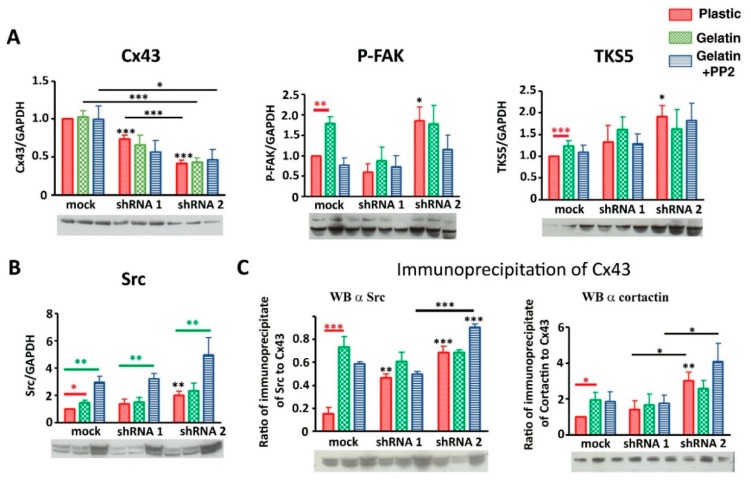
Protein (Cx43, P-FAK, TKS5 and Src) expressions and interactions in correlation with Cx43 expression. (**A**) Densitometric analysis of Cx43 (left), P-FAK (middle) and TKS5 (right) expression levels after normalization to GAPDH levels. (**B**) Densitometric analysis of Src protein expression level after normalization to GAPDH levels according to Cx43 expression level (N = 5). (**C**) Ratio of immunoprecipitation of Cx43 from U251 cells and immunoblotting of Src (middle) and cortactin (right) (N = 3). Red bar highlights differences due to gelatin culture condition and black bar highlights differences due to Cx43 expression diminution. Green bar highlights differences due to PP2 exposition. * *p* < 0.05; ** *p* < 0.01 and *** *p* < 0.001. Values shown as mean ± SEM (see Supplementary Table S1).

To decipher molecular mechanisms linking Cx43 to invadopodia formation and function, we estimated whether members of the Cx43 interactome present in invadopodia were affected by decreasing Cx43 expression. Indeed, in shRNA clones, we no longer observed an increase in expression of P-FAK, TKS5 and Src in the gelatin conditions compared to plastic, contrary to what was observed in mock cells (Figure 6A,B). Similarly, no interaction difference was observed between Cx43 and Src or cortactin whatever the culture conditions (Figure 6C).

**Figure 7:**
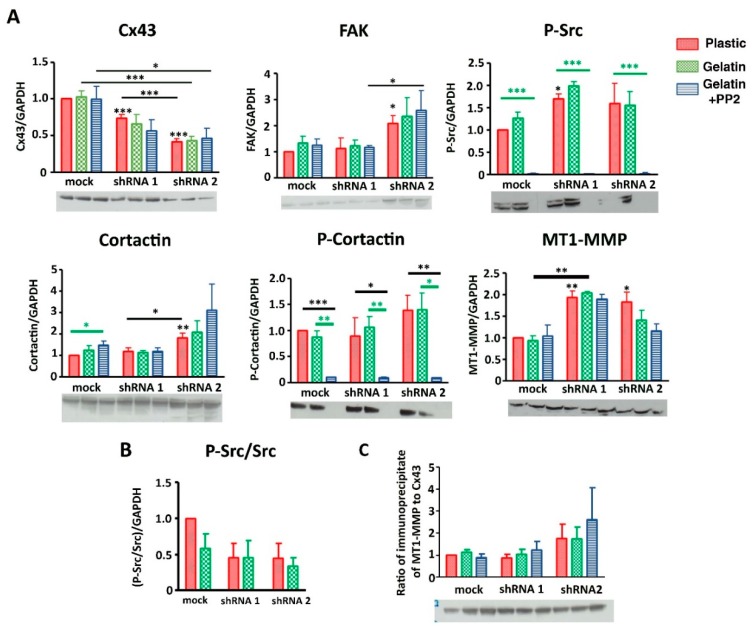
Protein (Cx43, FAK, P-Src, cortactin, P-cortactin, MT1-MMP) expressions and interactions in correlation with Cx43 expression. (**A**) First line: Densitometric analysis of Cx43 (left), FAK (middle) and P-Src (right) expression levels after normalization to GAPDH expression. Cx43 is significantly reduced in shRNA clones compared to mock cells cultivated on plastic (control, red), gelatin (0.2%, green) or gelatin + PP2 (5 µM, blue) conditions (N = 9). Second line: Densitometric analysis of cortactin (left), P-cortactin (middle) and MT1-MMP (right) protein expression level after normalization to GAPDH expression according to Cx43 expression level (N = 5). (**B**) Representation of Src activity by Src/P-Src ratio, (N = 5) (**C**) Ratio of immunoprecipitation of Cx43 from U251 cells and immunoblotting of MT1-MMP (N = 3). Red bar highlights differences due to gelatin culture condition and black bar highlights differences due to diminution of Cx43 expression. Representative blots are presented under all densitometric analysis. * *p* < 0.05; ** *p* < 0.01 and *** *p* < 0.001. Values shown as mean ± SEM (see Supplementary Table S1).

Moreover, we observed that the expression of key proteins in the formation of invadopodia increases after decreasing expression of Cx43 for shRNA 2 clone, in plastic culture (P-FAK, TKS5 and Src, Figure 6A,B; for FAK, P-Src, Cortactin and MT1-MMP, Figure 7A). No change was observed for Cx43/MT1-MMP interaction (Figure 7C).

So when Cx43 expression is decreased (shRNA clones), its interaction level with Src and cortactin was similar whatever the culture conditions (Figure 6C). This suggests that this interaction (Cx43/Src or Cx43/cortactin) is independent of gelatin stimulation, meaning that, instead of being transient, this interaction becomes permanent in shRNA cells contrary to mock cells. This transient interaction between Cx43 and Src or cortactin appears to be crucial for invadopodia formation.

Furthermore, by measuring the levels of Src phosphorylated at Tyr-416 (Y416 c-Src) compared to total Src (estimation of Src kinase activity), no difference was observed in mock cells whatever the culture conditions or shRNA cells (Figure 7B). Similarly, P-cortactin/total cortactin or P-FAK/total FAK were not changed in mock or shRNA cells (data not shown). Therefore, Src or cortactin activity is not responsible for the difference in invadopodia formation observed when Cx43 expression is decreased.

### 3.7. Evidence That Cx43 Hemichannels Inhibit Formation of Invadopodia

**Figure 8:**
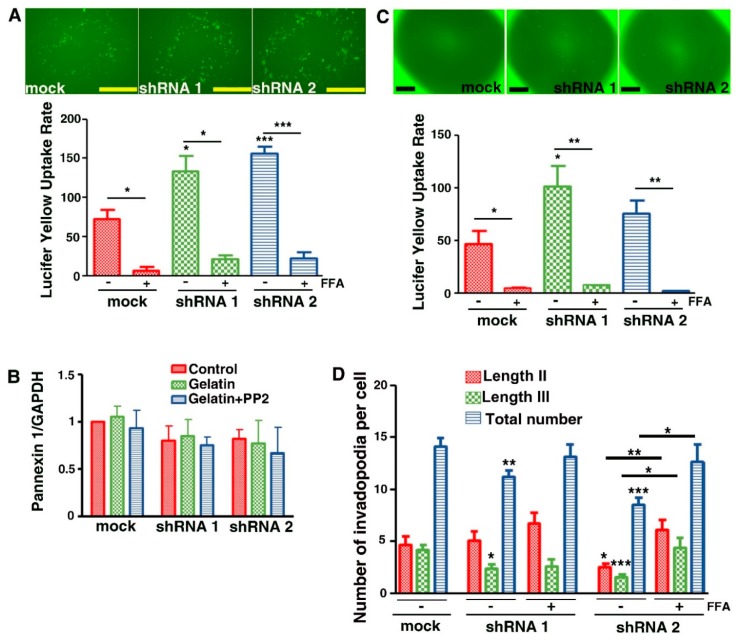
Correlation between increased hemichannel activity and decreased Cx43 expression. U251 cells were cultured on coverslips (**A**) or on inserts (**C**) coated with gelatin for 8 h. Lucifer Yellow (0.5 mg/mL) was added on coverslips or below the inserts permitting its uptake in cells via Cx43 hemichannels. For inserts, only Cx43 hemichannels in invadopodia were accessible to Lucifer Yellow. To determine the specific involvement of Cx43 hemichannel, a Cx inhibitor, flufenamic acid (FFA; 100 µM), was used. (**A**) Top panel: Representative records of Lucifer Yellow uptake by U251 cells cultured on coverslips coated with gelatin (N = 5) (Scale bar: 50 µm). Bottom panel: Quantification of Lucifer Yellow uptake depending on Cx43 expression level. (**B**) Densitometric analysis of Pannexin 1 (Panx1) expression levels after normalization by GAPDH expression. Panx1 expression is not significantly modulated in shRNA clones compared to mock cells on plastic (control, red), gelatin (0.2%, green) or gelatin + PP2 (5 µM, blue) conditions (N = 3). The increase in Lucifer Yellow uptake which is observed is therefore due to Cx43 decrease and not to Panx1. (**C**) Top panel: Representative record of Lucifer Yellow uptake of U251 cells cultured on inserts coated with gelatin (N = 5) (Scale bar: 50 µm). Bottom panel: Quantification of Lucifer Yellow uptake depending on Cx43 expression level. (**D**) Medium (red), long (green) and total number of invadopodia (blue) per cell was determined according to Cx43 expression level and in presence or not of FFA. Medium invadopodia correspond to the Assembly stage of invadopodia formation (Length II) and long invadopodia correspond to Elongation/Maturation stage of invadopodia formation (Length III). * *p* < 0.05; ** *p* < 0.01 and *** *p* < 0.001. Values shown as mean ± SEM (see Supplementary Table S1).

Since Cx43 may exhibit hemichannel activity when not involved in the formation of gap junctions, we studied hemichannel activity in Cx43 shRNA clones by the functional uptake of the fluorescent tracer Lucifer Yellow (LY) (Figure 8A). Unexpectedly, greater hemichannel activity was observed when the total Cx43 levels were reduced as compared to mock cells (Figure 8A). Dye uptake was inhibited by flufenamic acid (FFA), an inhibitor of Cx43 hemichannel and channel functions, suggesting that LY was entering the cells via Cx43 hemichannels. To minimize the possibility that dye uptake in Cx43 knockdown cells was mediated by elevated levels of Panx1 channels, we excluded this hypothesis by observing that Panx1 levels were the same in shRNA clones and mock cells (Figure 8B). The higher LY uptake is probably the consequence of increased Cx43 hemichannel activity, since we also observed higher ATP release in shRNA clones compared to mock cells (data not shown). Taken together, these results indeed suggest that the activity of hemichannels is inversely correlated with the expression of Cx43.

Co-immunoprecipitation showed an increased Cx43/Src interaction when Cx43 expression is decreased (Figure 6C). This suggests that increases in hemichannel activity and in Cx43/Src interaction are correlated in shRNA cells.

In further assessments, we examined whether Cx43 hemichannel activity is present in invadopodia. To accomplish this, mock and shRNA cells were seeded on porous filters (1 µm diameter) coated with gelatin that is impermeable to LY. Therefore, LY was added underneath the filter, allowing the fluorescent molecule to be in direct contact with the extremity of invadopodia crossing the gelatin and the filter. In such conditions, LY uptake occurred more readily in shRNA clones compared to mock cells (Figure 8C). Moreover, dye uptake was inhibited by FFA suggesting that Cx43 hemichannels were responsible for LY uptake (Figure 8C). These findings show that Cx43 exhibits higher hemichannel activity in Cx43 depleted cells that could prevent efficient formation and growth of invadopodia. Moreover, since no difference in hemichannel activity was seen between the mock cells and shRNA 2 cells, it was probably due to the shorter length of invadopodia in shRNA 2 cells at the times tested. Finally, the incubation of cells with hemichannel inhibitor FFA resulted in a similar number and length of invadopodia in shRNA clones and mock cells (Figure 8D).

## 4. Discussion

In cancer, invadopodia are cellular protrusions allowing ECM degradation that facilitates local invasion by tumor cells. Previous studies showed that invadopodia are made of protein networks interacting with actin filaments. The stages of invadopodia formation are highly regulated and require a series of protein phosphorylation and protein–protein interaction events [24,36]. Understanding how these structures are formed is of interest in therapeutics, if one is to design drugs to circumvent the invasion capacity and tumor recurrence. To investigate the role of Cx43 in these processes, we studied invadopodia formation and function in the invasive U251 human glioblastoma cell line. In a previous work, using a Cx43 knockdown approach, we observed that the invasive capacity of these cells was positively correlated with Cx43 expression level [32]. This correlation was in agreement with studies showing Cx43 involvement in glioma cell migration and invasion [2,3,4].

Here, we used immunolabeling and imaging approaches to demonstrate that glioblastoma cells digest gelatin at sites of invadopodia (ventral protrusions containing actin, cortactin, MT1-MMP and TKS5) and at FAs at the leading edge of the cells (containing actin, cortactin and P-FAK) (Figure 1 and Figure S1). By such approaches, for the first time, we show that Cx43 is localized in invadopodia at each stage of their growth (Figure 2, Figure 3 and Figure S2). Moreover, we also reveal that diminished level of Cx43 is correlated with a decreased in invadopodia formation and function. Indeed, the reduction in Cx43 expression induces a diminished number of lengths II and III invadopodia and also a decreased MMP2 proteolytic activity (Figure 5 and Figure S5).

Our studies support a role for Cx43 at the site of invadopodia formation, through a possible complex protein network (Figure 4). By co-immunoprecipitation, we found that Cx43 dynamically interacts with Src and cortactin (Figure 4D). However, no interactions between Cx43 and P-Src, P-cortactin or TKS5 were detected. When the cells are grown on gelatin (ECM mimetic stimulation), Src, P-FAK and TKS5 expressions (Figure 4A,B) and Cx43 interaction with specific partners (cortactin, Src; Figure 4D) were facilitated. However, when Cx43 was knocked down, this phenomenon was not observed (Figure 6). ECM is known to act on integrin protein activation or on cell surface protein-integrin complexes. This may suggest that in mock cells, the presence of gelatin directs cells toward adhesion through the stimulation of FAs, as shown by the increased level of P-FAK. It also increased invasion capacities by inducing the formation of invadopodia, except in Cx43 knock down cells.

**Figure 9:**
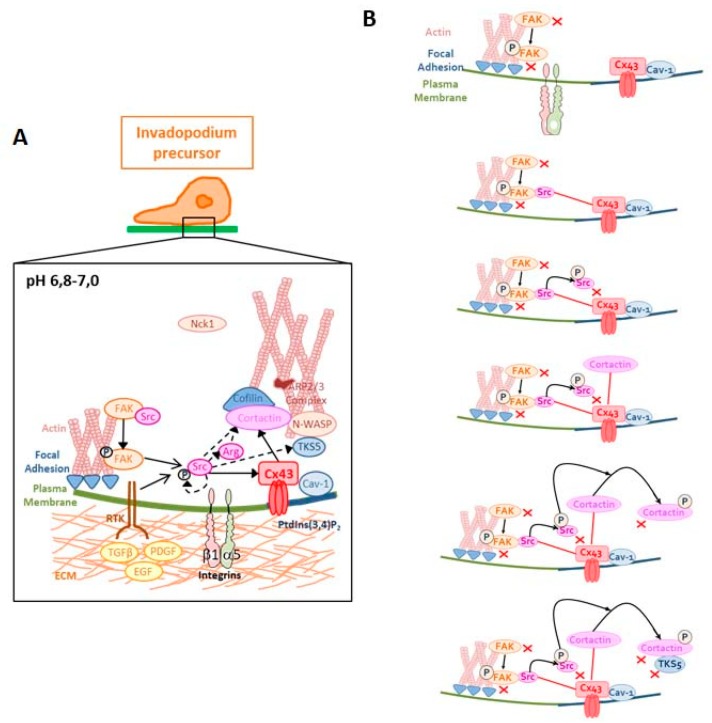
Possible role of Cx43 in invadopodia formation**.** Each stage of invadopodia formation is described in two parts: the cancer cell with localization of invadopodia structure (**A**) and a hypothetic description of protein interactions occurring during the invadopodia formation and growth (**B**). See Discussion for details.

During invasion, FAs are first stimulated to permit invadopodia formation. In FAs, the ligation of integrins to ECM provokes FAK autophosphorylation, permitting its interaction with Src [24]. The subsequent phosphorylation of FAK by Src (P-FAK) activates Src/P-FAK complex which phosphorylates, in turn, paxillin and p130CAS [37,38]. These events permit the FA assembly/disassembly that is essential for cell migration [39]. Our results show that Cx43 is not colocalised with P-FAK and FAK (Figure 2) and is absent in FAs, even though others have shown Cx43 to interact indirectly with integrin-α5, via protein 14-3-3θ [40], which is localized in invadopodia [41] (Figure 9). Moreover, it is known that Cx43 associates with other key elements of invadopodia which include cortactin, cofilin 1, caveolin-1, actin filaments or tubulin [30,31]. In invasive cells, in which invadopodia occur, a switch allows Src to be released from FAs to bind other substrates (cortactin, TKS5, caveolin-1, Cx43) necessary for invadopodia formation [28]. When Src is released from FA, rapid and transient interactions occur between Src and Cx43 [42] leading to Cx43 phosphorylation on Y247 (Figure 9). Once Cx43 becomes phosphorylated, this may regulate its interaction with other binding partners, such as ZO-1 and tubulin [43,44]. After Src autophosphorylation, Cx43 does not interact anymore with phosphorylated Src. This disrupted interaction could allow the phosphorylation of cortactin by Src, because of the proximity of these two proteins (Src and cortactin) favored by their interaction with Cx43. Once phosphorylated, the Src/cortactin complex does not seem to be linked with Cx43 anymore, permitting its proximity with a TKS5 complex (containing N-WASP, Nck1, Arp2/3 complex necessary for actin polymerization) (Figure 9). Therefore, we hypothesize that Cx43 has an effect on the rate of invadopodia formation by exerting a role in interacting proteins that enhances proximity between FA and invadopodia. In other words, Cx43 could facilitate interactions between Src and cortactin-cofilin complex. Indeed, when Cx43 expression is inhibited, cells would no longer have the ability to activate all processes (FA and invadopodia formations). In such conditions, a lower release and phosphorylation of partners involved in invadopodia formation—and, thus, decreased actin polymerization—would be observed (Figure 9).

Surprisingly, we found that densely plated U251 cells formed numerous gap junctions but less invadopodia (Figure S3). At a first glance, this appears contradictory with previous observations, showing that gap junctional intercellular communication (GJIC) increases glioma cell invasion. However, this was observed in the case of heterocellular GJIC between glioma and stroma cells [4,32] and homocellular GJIC between glioma cells favoring collective cell migration [45]. Both of these cases are different from the single cell migration/invasion examined in our current study. Indeed, Cx43 has minimal opportunity to form GJIC in sparse cultures and is then more available to potentially participate in invadopodia formation.

Intriguingly, decreased levels of Cx43 in U251 cells correlate with increased cellular levels of Cx43 hemichannel activity, even within invadopodia (Figure 8). So far, no study has shown that reducing Cx43 expression increases their hemichannel activity. However, new data have highlighted the role of the Cx43 Ct domain in the regulation of Cx43 hemichannel activity. Indeed, the recruitment of Src on this Ct domain leads to an increased activity of Cx43 hemichannels. Moreover, the use of negative regulators of Src causes the inhibition of Cx43 hemichannels [46,47]. In our results, we observed a significantly higher interaction between Cx43 and Src in shRNA cells compared to mock cells (Figure 6C). This Cx43/Src interaction could therefore explain the increased hemichannel activity in those cells.

In addition, hemichannel activity seems to have a negative effect on invadopodia formation. Indeed, its inhibition (FFA) induced a number of invadopodia similar to that observed in the mock cells (Figure 8D). At the invadopodia level, the function of Na^+^/H^+^ exchanger—NHE1—has been shown to be important for invadopodia formation and function, mostly in breast cancer cells [36,48,49]. Moreover, it was shown that NHE1 protein interacts with Cx43 [50]. NHE1 allows the output of H^+^ ions, resulting in the acidification of the extracellular microenvironment which favors MMPs activation. In parallel, in those conditions, the cytosol becomes alkaline, inducing cortactin and cofilin separation that is essential for actin polymerization and invadopodia formation [51]. After reducing Cx43 expression, we observed an increased hemichannel activity associated with decreased MMP2 activity (Figure S5) and invadopodia formation (Figure 8D). Therefore, it would be possible that the opening of Cx43 hemichannels occurring in Cx43 knockdown cells would prevent both the activation of MMP2 in ECM and invadopodia formation by permitting re-entry of H^+^ in the cytosol (Figure 9).

Our results show, for the first time, that a gap junction protein, Cx43, is localized in the invadopodia of glioblastoma-derived cells—such as U251 cells—and is involved in their formation and function. Together with previous data, Cx43 may play a role in the invasion process of U251 glioblastoma cells, by exerting two different functions. As an interacting protein, Cx43 would allow rapid interaction between two major partners involved in invadopodia formation—Src and cortactin. Moreover, Cx43 hemichannel activity seems to have an inhibitory role in invadopodia elongation and function, by its action on actin polymerization, supported by cortactin-cofilin complex, on one hand, and by its indirect action on MMP2 activity present in invadopodia, on the other.

In conclusion, our data obtained on U251 cells are a first step that has to be extended to other glioblastoma cells in order to understand the involvement of Cx43 in invadopodia formation. Such understanding might help developing therapies to prevent cancer cell invasion. If verified at a wider scale, the truncation of the Cx43 Ct domain could then be a way to prevent its interacting role necessary for invadopodia formation, without perturbing its ability to establish GJIC.

## Supplementary Materials

The following are available online at https://www.mdpi.com/2073-4409/9/1/117/s1, Figure S1: U251 cells are able to form invadopodia, Figure S2: U251 cells form invadopodia containing Cx43, Figure S3: Invadopodia formation and maturation are reduced when U251 mock cells are cultured at high density, Figure S4: Type 2 Matrix Metalloprotease (MMP2) decreases with Cx43 expression level, Figure S5: Specific knockdown of Cx43 expression in U251 cells, Table S1: Detailed data from Figure 4, Figure 5, Figure 6, Figure 7 and Figure 8.

## Abbreviations

Cx43	Connexin43
Ct	Carboxy-terminus
ECM	Extracellular matrix
FA	Focal adhesion
FAK	Focal adhesion kinase
FFA	Flufenamic acid
FG-gelatin	Fluorescent green gelatin
GJIC	Gap junctional intercellular communication
LY	Lucifer Yellow
MMP	Matrix metallo-proteinase
MT1-MMP	Membrane-type 1 matrix metalloproteinase
N-WASP	N-Wiskott–Aldrich syndrome protein
