# Supplementary material for: Involvement of the Gap Junction Protein, Connexin43, in the Formation and Function of Invadopodia in the Human U251 Glioblastoma Cell Line
Source: Cells. 2020 Jan 3;9(1):117. doi: 10.3390/cells9010117 (PMC7017254; doi:10.3390/cells9010117)
Supplement: Supplementary file 1 [file cells-09-00117-s001.pdf]

Table: Supplementary table: Detailed data from figures 4, 5, 6, 7 and 8.

**Figure 5**	mock	shRNA 1	shRNA 2
Percentage of cells presenting gelatin degradation due to the presence of invadopodia (Fig. 5C)	28.32 ± 1.93%	19.28 ± 1.75%	9.45 ± 1.84%
Total number of invadopodia per cell (Fig. 5D)	14.5 ± 1.1	9.1 ± 0.7	8.7 ± 0.7
Number of long and medium invadopodia per cell (Fig. 5E)	Medium 3.5 ± 0.5<br>Long 3.6 ± 0.3	Medium 3.3 ± 0.3<br>Long 1.5 ± 0.2	Medium 2.0 ± 0.2<br>Long 1.6 ± 0.3
**Figure 4/6/7**	mock	shRNA 1	shRNA 2
Western-blot			
Cx43 (Fig. 4A,6A ; N=9), plastic	1	0.74 ± 0.06	0.43 ±0.04
Cx43 (Fig. 4A,6A ; N=9), gelatin	1.03 ± 0.09	0.69 ± 0.14	0.45 ± 0.06
Cx43 (Fig. 4A,6A ; N=9), gelatin+PP2	1.01 ± 0.09	0.61 ± 0.16	0.47 ± 0.14
FAK (Fig. 4A,7A; N=4), plastic	1	1.13 ± 0.39	2.08 ± 0.3
FAK (Fig. 4A,7A; N=4), gelatin	1.33 ± 0.24	1.22 ± 0.22	2.36 ± 0.71
P-FAK (Fig. 4A,6A; N=4), plastic	1	0.93 ± 0.23	1.86 ± 0.32
P-FAK (Fig. 4A,6A; N=4), gelatin	1.78 ± 0.16	1.02 ± 0.3	1.87 ± 0.66
Src (Fig. 4B,6B ; N=5), plastic	1	1.37 ± 0.35	2 ± 0.27
Src (Fig. 4B,6B; N=5), gelatin	1.45 ± 0.16	1.74 ± 0.42	2.32 ± 0.55
Src (Fig. 4B,6B ; N=5), gelatin+PP2	2.97 ± 0.4	3.2 ± 0.41	4.96 ± 1.1
P-Src (Fig. 4B,7A ; N=3), plastic	1	1.7 ± 0.1	1.59 ± 0.45
P-Src (Fig. 4B,7A; N=3), gelatin	1.26 ± 0.13	1.99 ± 0.09	1.55 ± 0.31
P-Src (Fig. 4B,7A ; N=3), gelatin+PP2	0.02 ± 0.01	0.01 ± 0.001	0.03 ± 0.01
Immunoprecipitation			
Src (Fig. 4B,6C ; N=3), plastic	0.15 ± 0.05	0.47 ± 0.03	0.69 ± 0.05
Src (Fig. 4B,6C ; N=3), gelatin	0.74 ± 0.09	0.61 ± 0.08	0.69 ± 0.02
Western-blot			
Cortactin (Fig. 4C,7A ; N=4), plastic	1	1.19 ± 0.15	1.83 ± 0.21
Cortactin (Fig. 4C,7A ; N=4), gelatin	1.26 ± 0.21	1.13 ± 0.08	2.10 ± 0.50
P-Cortactin (Fig. 4C,7A; N=3), plastic	1	0.89 ± 0.34	1.39 ±0.28
P-Cortactin (Fig. 4C,7A; N=3), gelatin	0.87 ± 0.12	1.06 ± 0.19	1.39 ± 0.32
P-Cortactin (Fig. 4C,7A; N=3), gelatin+PP2	0.1 ± 0.09	0.1 ± 0.02	0.1 ± 0.09
TKS5 (Fig. 4D,6A; N=4), plastic	1	1.33 ± 0.53	1.91 ± 0.25
TKS5 (Fig. 4D,6A; N=4), gelatin	1.35 ± 0.03	1.61 ± 0.29	1.63 ± 0.43
MT1-MMP (Fig. 4D,7A ; N=3), plastic	1	1.94 ± 0.15	1.84 ± 0.22
MT1-MMP (Fig. 4D,7A ; N=3), gelatin	0.94 ± 0.11	2.04 ± 0.02	1.42 ± 0.22
Immunoprecipitation			
Cortactin (Fig. 4C,6C ; N=5), plastic	1	1.43 ± 0.45	3.02 ± 0.49
Cortactin (Fig. 4C,6C ; N=5), gelatin	1.95 ± 0.42	1.66 ± 0.62	2.57 ± 0.44
MT1-MMP (Fig. 4D,7C ; N=4), plastic	1	0.86 ±0.17	1.74 ±0.65
MT1-MMP (Fig. 4D,7C ; N=4), gelatin	1.13 ± 0.10	1.04 ± 0.23	1.73 ± 0.52
**Figure 8**	mock	shRNA 1	shRNA 2
Quantification LY uptake rate on coverslips			

Ctr (Fig. 8A ; N=5)	72 ± 12.76	133.6 ± 20.06	156 ± 8.67
+FFA (Fig. 8A ; N=5)	6 ± 5	21.5 ± 4.5	22.5 ± 7.5
WesternBlot Panx1			
Panx1 (Fig. 8B; N=3), plastic	1	0.8 ± 0.16	0.82 ±0.1
Panx1 (Fig. 8B; N=3), gelatin	1.05 ± 0.11	0.85 ± 0.17	0.77 ± 0.24
Panx1 (Fig. 8B; N=3), gelatin+PP2	0.93 ± 0.19	0.75 ± 0.09	0.67 ± 0.27
Quantification LY uptake rate on insert			
Ctr (Fig. 8C ; N=5)	46.38 ± 12.63	101.3 ± 19.52	75.38 ±12.97
+FFA (Fig. 8C ; N=5)	4.5 ±0.65	7.25 ± 0.85	2 ± 0.41
Quantification of invadopodia per cell			
Length II (Fig. 8D ; N=5)	4.63 ± 0.80	5.06 ± 0.86	2.50 ± 0.31
Length II + FFA (Fig. 8D ; N=5)	-	6.73 ± 0.97	6.06 ± 0.86

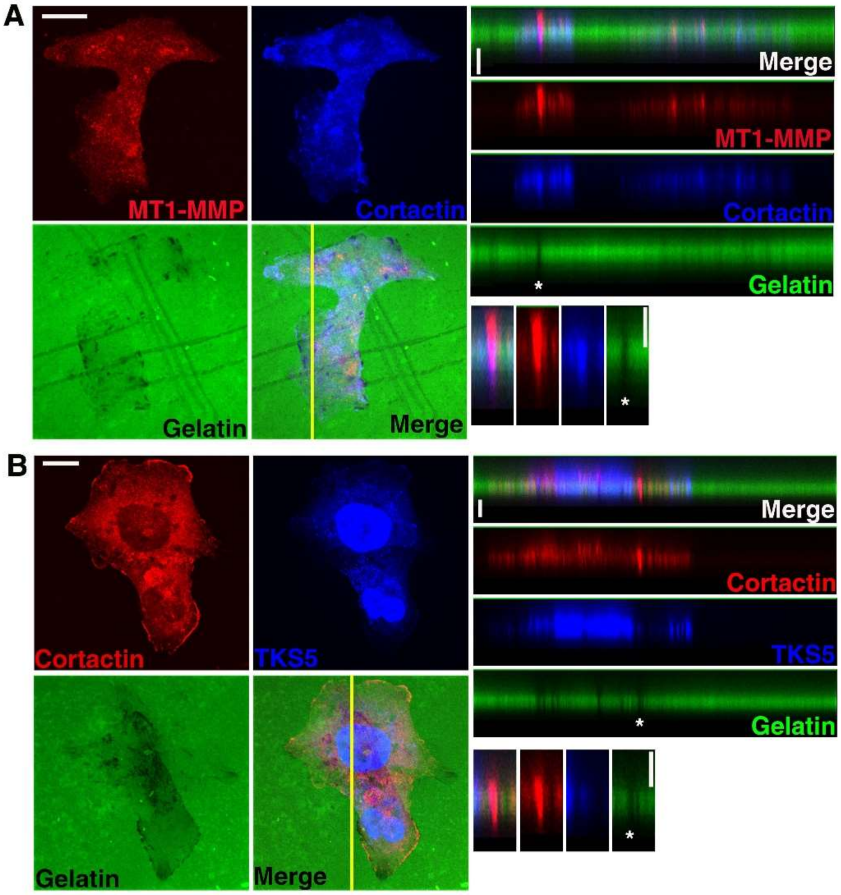

Figure S1: U251 cells are able to form invadopodia. Cells were cultured on coverslips with FG-gelatin (1.5 x 10^4 cells/mL) and observed by confocal microscopy in *xy* dimension. After 5 hours, localization of (**A**) MT1-MMP (Red) and cortactin (Blue) or (**B**) cortactin (Red) and TKS5 (Blue) was determined by indirect immunofluorescence. Invadopodia formation (*) is observed. Left panel is *xy* images, right top panel is *xz* images and right bottom panel is *xz*

enlargement of regions of interest (N=10). The yellow line in *xy* images is the axis of *xz* dimension. (Scale bars: 20µm on *xy* plan and 2µm on *xz* plan).

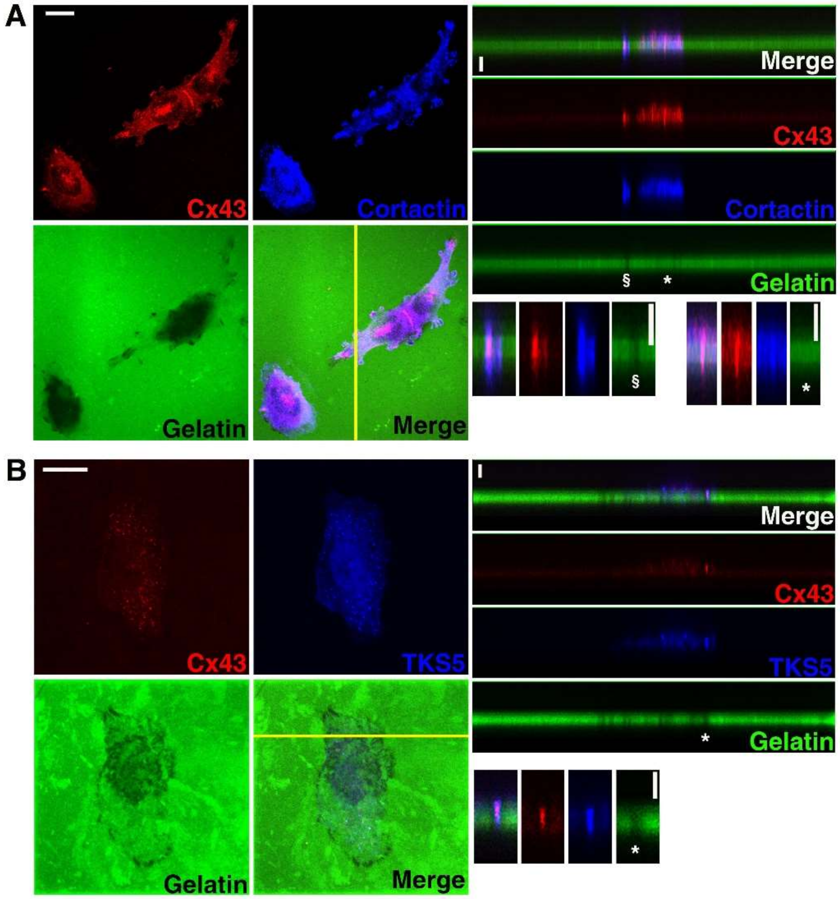

Figure S2: U251 cells form invadopodia containing Cx43. Cells were cultured on coverslips coated with FG-gelatin (1.5 x 10^4 cells/mL) and observed by confocal microscopy in *xy* dimension. After 5 hours, localization of (**A**) Cx43 (Red) and cortactin (Blue) or (**B**) Cx43 (Red) and TKS5 (Blue) was determined by indirect immunofluorescence. Invadopodia formation (*) and focal adhesions (§) were observed. Each left panel is *xy* images, right top panel is *xz* images and right bottom panel is *xz* enlargement of regions of interest (N=10). The yellow line in *xy* images is the axis of *xz* dimension. (Scale bar: 20µm on xy plan and 2µm on xz plan).

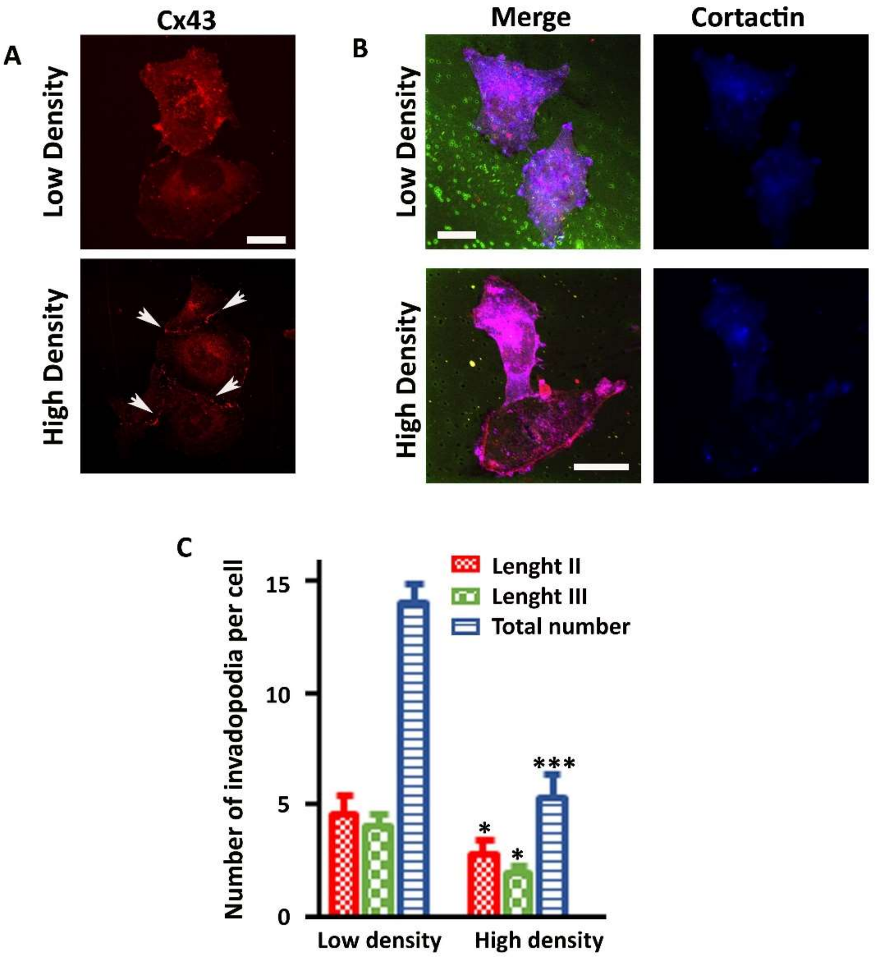

Figure S3: Invadopodia formation and maturation are reduced when U251 mock cells are cultured at high density. (**A**,**B**) Confocal microscopy of U251 mock cells cultured on inserts coated with FG-gelatin at low (top panel) or high (bottom panel) density. (**A**) Cx43 (Red), (**B**) cortactin (Blue) is detected by indirect immunofluorescence and F-actin (Red) by phalloïdin. Cells were observed in *xy* dimension (N=10) (Scale bar: 20µm). (**C**) Length II, length III and total number of invadopodia per cell was determined according to cell density culture condition. Length II invadopodia correspond to Assembly stage of invadopodia formation and length III invadopodia correspond to Elongation/Maturation stage of invadopodia formation. At low density, total number per cell of length II invadopodia is 4.63 ± 0.80; length III invadopodia, 4.13 ± 0.51 and total number of invadopodia, 14.06 ± 0.82. At high density, the total number per cell of length II invadopodia is 2.89 ± 0.56 per cell; length III invadopodia, 2.0 ± 0.37 and

total number of invadopodia, 5.36 ± 1.04. *P < 0.05 and ***P < 0.001. Values shown as mean ± SEM.

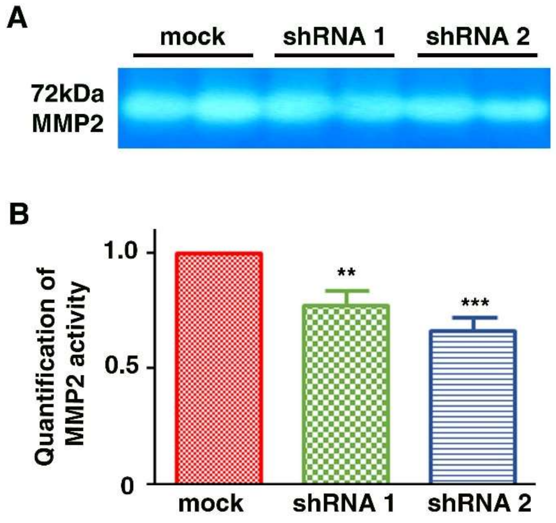

Figure S4: Type 2 Matrix Metalloprotease (MMP2) decreases with Cx43 expression level. (**A**) Representative gelatin zymography gel after 72 hours of incubation. U251 mock cells (left), sh-RNA 1 (middle) or sh-RNA 2 (right) supernatant was 10-fold concentrated before electrophoresis. (**B**) Densitometric evaluation of gelatin zymography according to Cx43 expression level (N=7). Mock cells: 1.00; sh-RNA 1: 0.78 ± 0.06; sh-RNA 2: 0.66 ± 0.06. **P < 0.01 and ***P < 0.001. Values shown as mean ± SEM.

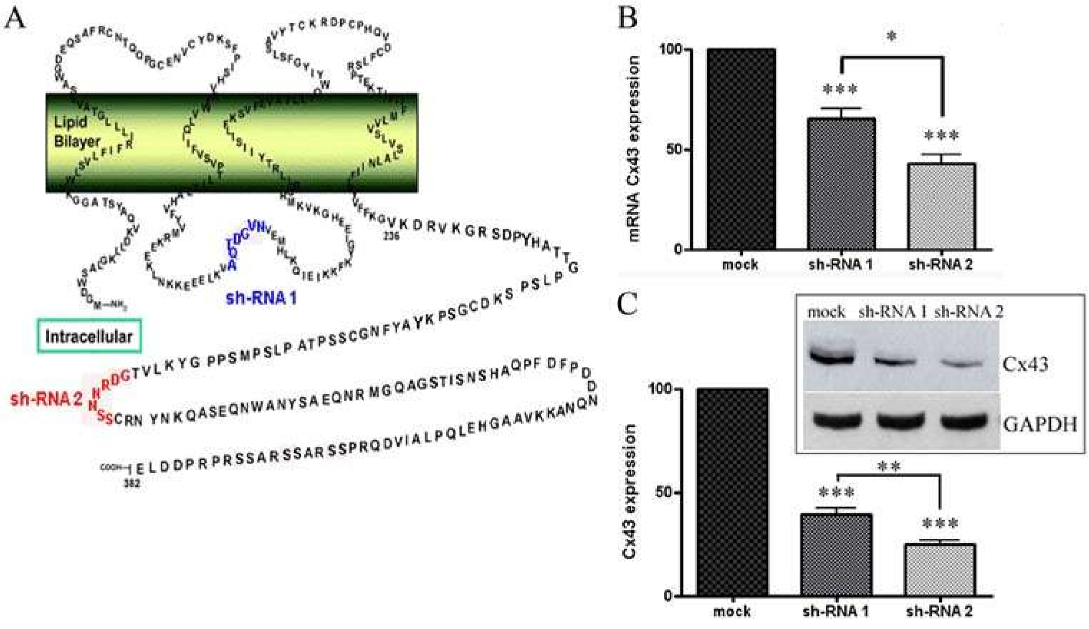

Figure S5: Specific knockdown of Cx43 expression in U251 cells. A) Schematic representation of Cx43 domains (intracellular loop for shRNA 1 and carboxy-terminal for shRNA 2) targeted by sh-RNAs. B) Cx43 mRNA was monitored by quantitative real-time RT-PCR. Values of 4 independent experiments done in triplicate are expressed as percentage of mock after normalization by GAPDH expression. The level of Cx43 mRNA is significantly reduced in shRNA 1 and shRNA 2 clones compared to mock cells (shRNA 1: 65.4 $\pm$ 4.8 % and shRNA 2: 42.8 $\pm$ 4.5 %). C) *Up panel*: Representative immunoblot of Cx43, GAPDH is used as a loading control. *Down panel*: Densitometric analysis of Cx43 protein expression of 4 independent experiments after normalization by GAPDH expression. Cx43 is significantly reduced in shRNA 1 and shRNA 2 clones compared to mock (sh-RNA 1: 39.1 $\pm$ 3.0 % and sh-RNA 2: 24.7 $\pm$ 1.9 %).
